# Supplementary material for: Host factor prioritization for pan-viral genetic perturbation screens using random intercept models and network propagation
Source: PLoS Comput Biol. 2020 Feb 10;16(2):e1007587. doi: 10.1371/journal.pcbi.1007587 (PMC7034926; doi:10.1371/journal.pcbi.1007587)
Supplement: S1 Text — Description of methods we used for normalizing and integrating the data sets. (PDF) [file pcbi.1007587.s002.pdf]

S1 Text. Normalisation methods. We applied different combinations of normalization methods to get phenotypes that are comparable between the studies. In summary, we used the following methods for normalization:

- Logarithm. Compute the natural logarithm over every well-readout separately, such that a well's new readout y_{ij} is computed as $y_{ij}^{\text{norm}} = \log(y_{ij})$.
- Background correction. Substract the mean/median of a row/column from every well of every plate separately.
- Robust Z-scores. Substract by median and divide by median absolute deviation over every plate separately. The new readout is computed as $y_{ij}^{\text{norm}} = \frac{y_{ij} - \text{med}(\mathbf{Y})}{\text{mad}(\mathbf{Y})}$, where med$(\mathbf{Y})$ is the median of plate readouts and mad$(\mathbf{Y})$ is the median absolute deviation of plate readouts.
- Loess. Fits a locally-weighted polynomial regression model between well cell counts x_i and averaged well readout y_i separately on every plate using `R`'s `lowess` function. The procedure estimates a new readout $\hat{y}_i$ for every cell counts covariable x. The new normalized value is computed as $y_i^{\text{norm}} = y_i - \hat{y}_i$, i.e. the residual.
- B-scores. B-scores are computed using `R`'s `medpolish` function on every plate separately. Input to `medpolish` is a matrix $\mathbf{Y}$ where every element y_{ij} is the readout from the i-th row and j-th column from a plate. The new normalized value per row and column is computed as $y_{ij}^{\text{norm}} = \frac{r_{ij}}{\text{mad}(\mathbf{R})}$, where $\mathbf{R}$ are the computed residuals for every well ij and mad($\mathbf{R}$) is the median absolute deviation of the residuals.

The implementation of the described methods can be found in Supplement S1 Code.
