# Supplementary material for: Host factor prioritization for pan-viral genetic perturbation screens using random intercept models and network propagation
Source: PLoS Comput Biol. 2020 Feb 10;16(2):e1007587. doi: 10.1371/journal.pcbi.1007587 (PMC7034926; doi:10.1371/journal.pcbi.1007587)
Supplement: S2 Text — Description of creation of the synthetic data sets for stability analysis. (PDF) [file pcbi.1007587.s003.pdf]

S2 Text. Stability analysis. For validation of the stability of the rankings, we generate several different data sets that are parameterized by a noise variance σ^2, a virus count V, a replicate count R, a gene count G and screen count S. The steps to generate one data set for stability analysis are as follows"

1. Generate a random matrix of G columns and $10 \cdot G$ rows. Then sample a random undirected scale free network $\mathcal{G}$ of G nodes using the `barabasi.game` method from the `igraph`-package [1]. Next iterate over the set of edges from $\mathcal{G}$ and set every edge weight to a random value following a Beta distribution with shapes $\alpha = 2$ and $\beta = 1$ (the shape parameters and the underlying distribution were chosen arbitrarily and other distributions with non-negative support are equally suited). From this adjacency matrix we compute the correlation matrix $\mathbf{\Sigma}$.

2. Create standard normally distributed random vectors $\boldsymbol{\nu} \sim \mathcal{N}_V(\mathbf{0}, \mathbf{I})$, $\boldsymbol{\tau} \sim \mathcal{N}_S(\mathbf{0}, \mathbf{I})$ and $\boldsymbol{\gamma} \sim \mathcal{N}_G(\mathbf{0}, \mathbf{\Sigma})$.

3. Take all combinations $\mathcal{D} = (\boldsymbol{\nu} \times \boldsymbol{\tau} \times \boldsymbol{\gamma})$, i.e. take all combinations of elements $\nu_j \in \boldsymbol{\nu}$, $\tau_i \in \boldsymbol{\tau}$ and $\gamma_k \in \boldsymbol{\gamma}$ in a $(V \cdot S \cdot G \times 3)$-dimensional matrix where every row is a vector $(\nu_j, \tau_i, \gamma_k)$.

4. Replicate each row from the data set $\mathcal{D}$ R times.

5. Add i.i.d. Gaussian noise $\epsilon \sim \mathcal{N}(\mathbf{0}, \sigma^2\mathbf{I})$ to all rows independently.

[1] Csardi G, Nepusz T, et al. The igraph software package for complex network research. InterJournal, Complex Systems. 2006;1695(5):1–9.
