# Supplementary material for: Host factor prioritization for pan-viral genetic perturbation screens using random intercept models and network propagation
Source: PLoS Comput Biol. 2020 Feb 10;16(2):e1007587. doi: 10.1371/journal.pcbi.1007587 (PMC7034926; doi:10.1371/journal.pcbi.1007587)
Supplement: S3 Text — Description of creation of the synthetic data sets for predictability analysis. (PDF) [file pcbi.1007587.s004.pdf]

S3 Text. Analysis of predictive performance. A simulated data set that validates predictive performance is parameterized by parameters for the number of replicates R, the number of viruses V, and a noise variance σ^2. We use a fixed gene count of $G = 100$ and number of screening types $S = 5$. The steps to generate the simulated data are:

1. Generate a standard normally distributed random vector $\boldsymbol{\gamma} \sim \mathcal{N}_{90}(\mathbf{0}, \mathbf{I})$. Create random vectors $\boldsymbol{\gamma}_h \sim \mathcal{N}_5(\mathbf{0.5}, \mathbf{I})$ and $\boldsymbol{\gamma}_l \sim \mathcal{N}_5(-\mathbf{0.5}, \mathbf{I})$ and append these vectors to $\boldsymbol{\gamma}$. The two latter vectors represent the *true* hit genes, i.e., genes that have an impact on the life cycle of a group of pathogens. In the end the gene effect vector $\boldsymbol{\gamma}$ is a three-component mixture of Gaussians $f(x) = 0.9 \cdot f_0(x) + 0.05 \cdot f_h(x) + 0.05 \cdot f_l(x)$ where f_h and f_l represent the distributions readouts for anti-viral genes and pro-viral genes, respectively and f_0 is the distribution of readouts for null-genes, i.e., genes for which a transcript does not lead to changes in pathogen replication.

2. Create standard normal distributed random vectors $\boldsymbol{\nu} \sim \mathcal{N}_v(\mathbf{0}, \mathbf{I})$ and $\boldsymbol{\tau} \sim \mathcal{N}_s(\mathbf{0}, \mathbf{I})$.

3. Take all combinations $\mathcal{D} = (\boldsymbol{\nu} \times \boldsymbol{\tau} \times \boldsymbol{\gamma})$, i.e. take all combinations of elements $\nu_j \in \boldsymbol{\nu}$, $\tau_i \in \boldsymbol{\tau}$ and $\gamma_k \in \boldsymbol{\gamma}$ in a $(V \cdot S \cdot G \times 3)$-dimensional matrix where every row is a vector $(\nu_j, \tau_i, \gamma_k)$.

4. Replicate each row from the data set $\mathcal{D}$ R times.

5. Add i.i.d. Gaussian noise $\epsilon \sim \mathcal{N}(\mathbf{0}, \sigma^2\mathbf{I})$ to all rows independently.
