# Supplementary material for: Host factor prioritization for pan-viral genetic perturbation screens using random intercept models and network propagation
Source: PLoS Comput Biol. 2020 Feb 10;16(2):e1007587. doi: 10.1371/journal.pcbi.1007587 (PMC7034926; doi:10.1371/journal.pcbi.1007587)
Supplement: S4 Text — Description of the experimental protocol of the compound screens and data normalization. (PDF) [file pcbi.1007587.s005.pdf]

S4 Text. Compound screen data acquisition. Compounds were purchased from companies indicated below and dissolved according to the manufacturers instructions in DMSO.

HCV/DENV Huh7.5 cells ([1]) stably expressing Firefly luciferase (Huh7.5 Fluc) were seeded on white 96-well plates at 1x104 cells per well 16 hours prior to infection with the HCV reporter virus JcR2a (genotype 2a, [2]) and 16 to 24 hours prior to infection with a DENV reporter virus carrying a Renilla luciferase (DenR2a). For HCV, compounds were added with the virus at an MOI of 0.1 at time-point 0 hours or after virus removal at 8 hours post infection. For DENV,cCompounds were added with the virus at an MOI of 0.5. For both viruses, cells were lysed 72 hours later in 1x Promega Lysis Buffer (Promega, Mannheim, Germany), frozen at -80C, and luciferase activities were measured for 1 sec with D Luciferin with no filter (Firefly) and 10 sec with Coelenterazine with a 480 nm high-sense filter (Renilla) in a Mithras LB 943 plate luminometer (Berthold Technologies, Bad Wildbad, Germany).

CVB: Huh7 high passage cells were seeded in a 96-well plate at 5 x103 cells per well (next day 75% confluent) one day prior to infection. Compounds were added with CVB3 at 100 TCID50 per well. Cell viability was determined 24 hours post infection (using MTS assay according to the manufacturer protocol (Aqueous; Promega).

CHIKV/MERS: VeroE6 cells were seeded at a density of 5,000 cells/well in 96 well plates and HuH7 cells at 10,000 cells/well in clear 96 well plates. The next day, the culture medium was replaced with dilutions of the compounds in medium and Vero E6 cells were infected with CHIKV (100 pfu/well) and Huh7 cells with MERS-CoV (200 pfu/well). At 96 hpi for CHIKV and 72 hpi for MERS-CoV, a colorimetric viability assay was performed by adding 20 l/well of the CellTiter 96 AQueous One Solution Cell Proliferation Assay reagent (Promega). The reaction was stopped after 2-2.5 h by fixing the cells with 37% formaldehyde. The absorbance was measured at 495 nm in a Berthold Mithras LB 940 plate reader, and the values were expressed relative to uninfected (infection) or untreated (viability) samples.

Compound screen meta data.

Gene	Compound	Manufacturer	Article number/ID
CAMKK2	STO-609	Tocris	1551
CDK5R2	Alsterpaullone	Sigma-Aldrich	A4847
CSNK2B	Ellagic Acid	Tocris	3058
DGKE	Dioctanoylglycol	Tocris	0484
DUSP1	BCI hydrochlorid	Sigma-Aldrich	B4313
DYRK1B	AZ 191	Tocris	5232
PIK4CA	PI 93	Sigma-Aldrich	SML0546
PKN3	Quercetin Dihydrate	Sigma-Aldrich	Y0001009
PLK1	Ro 3280	Tocris	5968

[1] Blight KJ, McKeating JA, Rice CM. Highly Permissive Cell Lines for Subgenomic and Genomic Hepatitis C Virus RNA Replication. Journal of Virology. 2002;76(24):13001–13014.

[2] Reiss S, Rebhan I, Backes P, Romero-Brey I, Erfle H, Matula P, et al. Recruitment and Activation of a Lipid Kinase by Hepatitis C Virus NS5A Is Essential for Integrity of the Membranous Replication Compartment. Cell Host & Microbe. 2011;9(1):32–45.
