# Supplementary material for: Host factor prioritization for pan-viral genetic perturbation screens using random intercept models and network propagation
Source: PLoS Comput Biol. 2020 Feb 10;16(2):e1007587. doi: 10.1371/journal.pcbi.1007587 (PMC7034926; doi:10.1371/journal.pcbi.1007587)
Supplement: S5 Text — Description of the experimental protocol of the siRNA validation screen, data normalization and the statistical analysis. (PDF) [file pcbi.1007587.s006.pdf]

S5 Text. siRNA-knockdown of predicted host factors for HCV. In order to confirm the network analysis-predicted host factors UBC, PLCG1, and EP300, we infected Huh7.5 Fluc cells (Blight *et al.*) with our reporter virus JcR2a [1]. 16 hours prior to infection, we reverse transfected 6 pmol siRNA (final concentration 10 nM) in 5x104 cells in a 24 well format using RNAiMax (Thermo Fisher Scientific, Karlsruhe, Germany) following the manufacturers instructions. 72 hours after JcR2a infection, cells were lysed in 1x Passive Lysis Buffer (Promega, Mannheim, Germany) and luciferase activities were measured on a Mithras LB 943 plate luminometer (Berthold Technologies, Bad Wildbad, Germany). Renilla luciferase signals are normalized to Firefly luciferase. Three independent biological experiments have been conducted in triplicates each. We tested the normalized data of each siRNA (denoted EP300_1, EP300_2, UBC_1, UBC_2, PLCG1_1, PLCG1_2 and a positive control PI4K in Figure 5) against an AllStars negative control siRNA (Qiagen, Hilden, Germany) using a two-sided two-sample Wilxocon test, i.e. each test consisted of two samples of size 9 each. Respective scripts for data analysis can be found in Supplement S1 Code and the data in S1 Data.

[1] Blight KJ, McKeating JA, Rice CM. Highly Permissive Cell Lines for Subgenomic and Genomic Hepatitis C Virus RNA Replication. Journal of Virology. 2002;76(24):13001–13014.
