# Supplementary material for: Host factor prioritization for pan-viral genetic perturbation screens using random intercept models and network propagation
Source: PLoS Comput Biol. 2020 Feb 10;16(2):e1007587. doi: 10.1371/journal.pcbi.1007587 (PMC7034926; doi:10.1371/journal.pcbi.1007587)
Supplement: S6 Text — (PDF) [file pcbi.1007587.s007.pdf]

S6 Text. Details on estimation in linear mixed models. We briefly explain the single steps of the model. We start with the general vector notation of the conditional Gaussian (multi-level) mixed model from Equation (1):

$$\mathbf{y}_{vgt} \mid \mathbf{u}_{vgt} \sim \mathcal{N}\left(\mathbf{x}_{vgt}\beta + \mathbf{Z}_{vgt}\mathbf{u}_{vgt}, \sigma^2\mathbf{I}\right),$$

where $v = 1, \ldots, V$ are the different viruses, $g = 1, \ldots, G$ are genes and $t = 1, \ldots, T$ are different screen types. The vector $\mathbf{y}_{vgt}$ consists of all readouts that have been measured for virus v, gene g and screen type t, $\mathbf{u}_{vgt} = (\gamma_g,\ \delta_{vg},\ \zeta_t,\ \xi_{vt})^T$ is a vector of random effects, and $\mathbf{Z}_{vgt} = \mathbf{1}^{\text{len}(\mathbf{y}_{vgt})\times 4}$ (because of 4 random intercept terms) is a random effects design matrix. The random effects $\mathbf{u}$, the fixed effect β and the noise variance σ^2 are random variables or unknown constants of interest to be predicted, or estimated, respectively. When we collect the entire data set $(\mathbf{x}_{vgt}, \mathbf{y}_{vgt})$, i.e. all vectors for every combination of viruses, genes and screen types, we arrive at the general mixed model formulation which we need for estimation:

$$\mathbf{x} = \begin{pmatrix} \mathbf{x}_{1,1,1} \\ \vdots \\ \mathbf{x}_{v,g,t} \\ \vdots \\ \mathbf{x}_{V,G,T} \end{pmatrix}, \qquad \mathbf{y} = \begin{pmatrix} \mathbf{y}_{1,1,1} \\ \vdots \\ \mathbf{y}_{v,g,t} \\ \vdots \\ \mathbf{y}_{V,G,T} \end{pmatrix}, \qquad \mathbf{u} = \begin{pmatrix} \mathbf{u}_{1,1,1} \\ \vdots \\ \mathbf{u}_{v,g,t} \\ \vdots \\ \mathbf{u}_{V,G,T} \end{pmatrix}$$

It follows that when collecting all random effect design matrices $\mathbf{Z}_{v,g,t}$ we get a block diagonal matrix:

$$\mathbf{Z} = \text{blockdiag}\left(\mathbf{Z}_{1,1,1}, \ldots, \mathbf{Z}_{v,g,t}, \ldots, \mathbf{Z}_{V,G,T}\right)$$

Thus the general notation of the linear mixed model is

$$\mathbf{y} \mid \mathbf{u} \sim \mathcal{N}\left(\mathbf{x}\beta + \mathbf{Z}\mathbf{u}, \sigma^2\mathbf{I}\right),$$

where

$$\mathbf{u} \sim \mathcal{N}\left(\mathbf{0}, \mathbf{G}\right),$$

where $\mathbf{G}$ is an unknown covariance matrix to be estimated. Recent research such as [1] introduced efficient approaches to estimation of parameters β, σ^2 and $\mathbf{G}$ and prediction of $\mathbf{u}$ which go beyond the scope of this paper. We recall here the general approach which for instance can be found in [2]: From the formalisation above we can integrate out the random effects $\mathbf{u}$ which gives us the marginal model:

$$\mathbf{y} \sim \mathcal{N}\left(\mathbf{x}\beta, \sigma^2\mathbf{I} + \mathbf{Z}\mathbf{G}\mathbf{Z}^T\right),$$

We can now formalize an objective, the likelihood of the marginal model:

$$L(\beta, \mathbf{G}, \sigma^2) = P(\mathbf{y} \mid \beta, \mathbf{G}, \sigma^2)$$

For restricted maximum likelihood estimation we now treat β as random variable, too, e.g. as in an empirical Bayesian context with a flat prior, and marginalize it out:

$$L(\mathbf{G}, \sigma^2) = \int P(\mathbf{y} \mid \beta, \mathbf{G}, \sigma^2) d\beta$$

Estimation of parameters of the LMM is done in two steps: first we estimate $\mathbf{G}$ and σ^2 by maximizing the likelihood above numerically, for instance using a quasi-Newton method. Second we estimate β and $\mathbf{u}$ using Henderson's mixed model equations analytically as:

$$\hat{\beta} = \left(\mathbf{x}^t\mathbf{V}^{-1}\mathbf{x}\right)^{-1}\mathbf{x}^T\mathbf{V}^{-1}\mathbf{y}$$
$$\hat{\mathbf{u}} = \mathbf{R}\mathbf{U}^{-1}\mathbf{V}^{-1}\left(\mathbf{y} - \mathbf{x}\hat{\beta}\right)$$

where $\mathbf{V} = \mathbf{Z}\mathbf{R}\mathbf{Z}^T + \sigma^2\mathbf{I}$.

[1] Bates D, Mächler M, Bolker BM, Walker SC. Fitting linear mixed-effects models using lme4. Journal of Statistical Software. 2015;67(1):1–48.

[2] McCulloch CE, Searle SR. Generalized, Linear, and Mixed Models. Wiley Series in Probability and Statistics; 2001.
