# Supplementary material for: Host factor prioritization for pan-viral genetic perturbation screens using random intercept models and network propagation
Source: PLoS Comput Biol. 2020 Feb 10;16(2):e1007587. doi: 10.1371/journal.pcbi.1007587 (PMC7034926; doi:10.1371/journal.pcbi.1007587)

S1 Figure. Normalisation effect on readouts of plate controls. Comparison of plate readouts for positive and negative controls for the HCV kinome screen. Subfigure (a) shows the un-normalized control readouts. Negative and positive controls suffer from a high variance. (b) Normalizing the data plate-wise reduces the variance for the negative controls. The positive controls, however, still exert a high variation even after normalizing which could be due to stochastic knock down patterns.

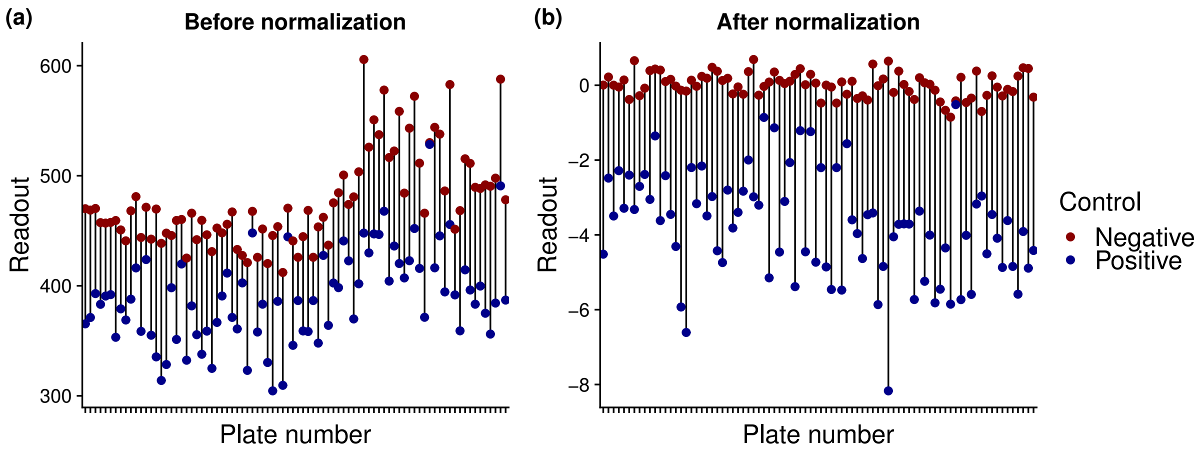

Supplement: S1 Fig — Comparison of plate readouts for positive and negative controls. (PDF) [file pcbi.1007587.s009.pdf]
