# Supplementary material for: Host factor prioritization for pan-viral genetic perturbation screens using random intercept models and network propagation
Source: PLoS Comput Biol. 2020 Feb 10;16(2):e1007587. doi: 10.1371/journal.pcbi.1007587 (PMC7034926; doi:10.1371/journal.pcbi.1007587)

S2 Figure. Normalisation effect on control distributions. Comparison between unnormalized and normalized control densities of the HCV kinome screen. For every positive control (green) and negative control (red) density functions are shown. Ideally the two mixture components are separated strongly. While before normalization in (a) the two components do not show a strong separation, the control distributions could be separated much better after normalisation (b).

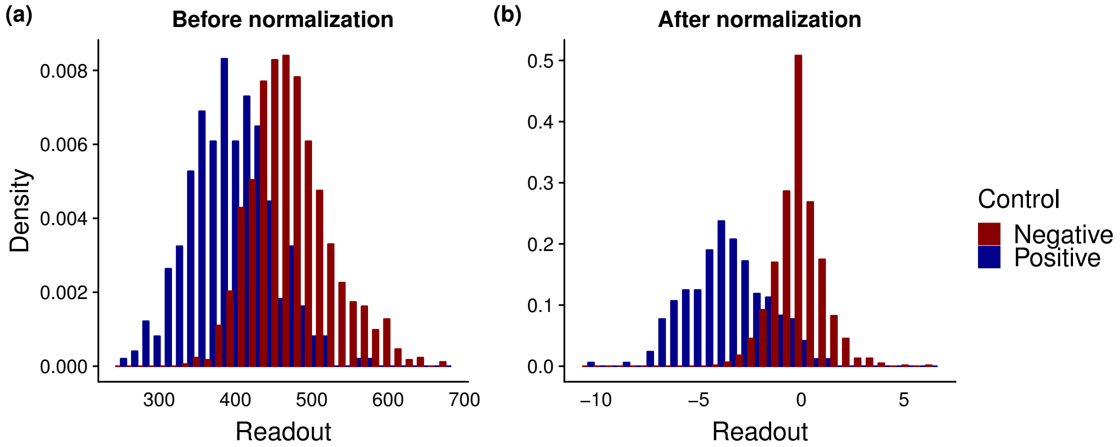

Supplement: S2 Fig — Comparison between unnormalized and normalized control densities. (PDF) [file pcbi.1007587.s010.pdf]
