# Supplementary material for: Host factor prioritization for pan-viral genetic perturbation screens using random intercept models and network propagation
Source: PLoS Comput Biol. 2020 Feb 10;16(2):e1007587. doi: 10.1371/journal.pcbi.1007587 (PMC7034926; doi:10.1371/journal.pcbi.1007587)

S3 Figure. Pathogen-specific gene hits. Every subplot shows the most significant genes for a virus using a false discovery rate threshold of 0.2. Using multiple testing correction on the pathogen-specific gene effects gives only a few significant genes all of which are viral dependency factors.

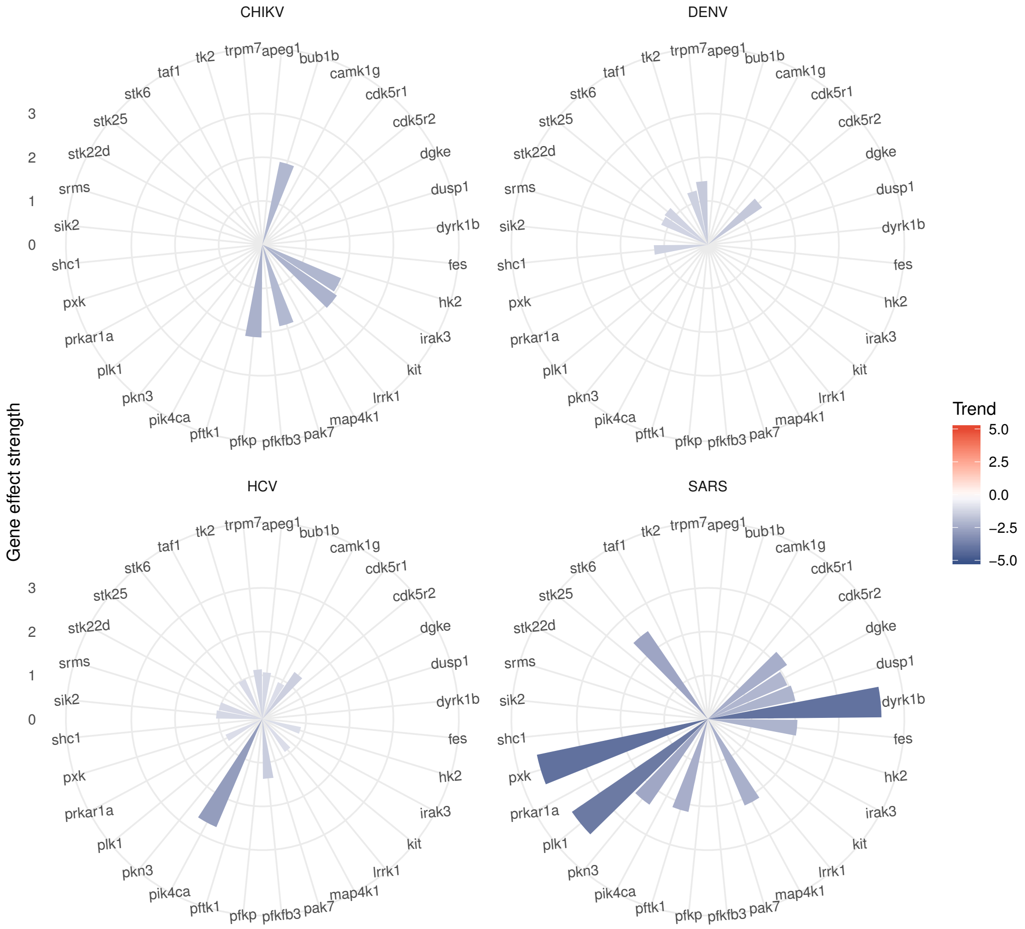

Supplement: S3 Fig — Visualization of the top pathogen-specific effect sizes. (PDF) [file pcbi.1007587.s011.pdf]
