# Supplementary material for: Host factor prioritization for pan-viral genetic perturbation screens using random intercept models and network propagation
Source: PLoS Comput Biol. 2020 Feb 10;16(2):e1007587. doi: 10.1371/journal.pcbi.1007587 (PMC7034926; doi:10.1371/journal.pcbi.1007587)

S4 Figure. Predictive performance on simulated and biological data. The plots show the performance of our hierarchical model and PMM. (a) Our model shows a lower mean squared error for all error variances $\sigma^2 = 1$, $\sigma^2 = 2$ and $\sigma^2 = 5$. (b) Increasing the number of viruses lowers the mean squared errors in both models marginally. For all benchmarks our model had a minor, non-significant advantage over PMM.

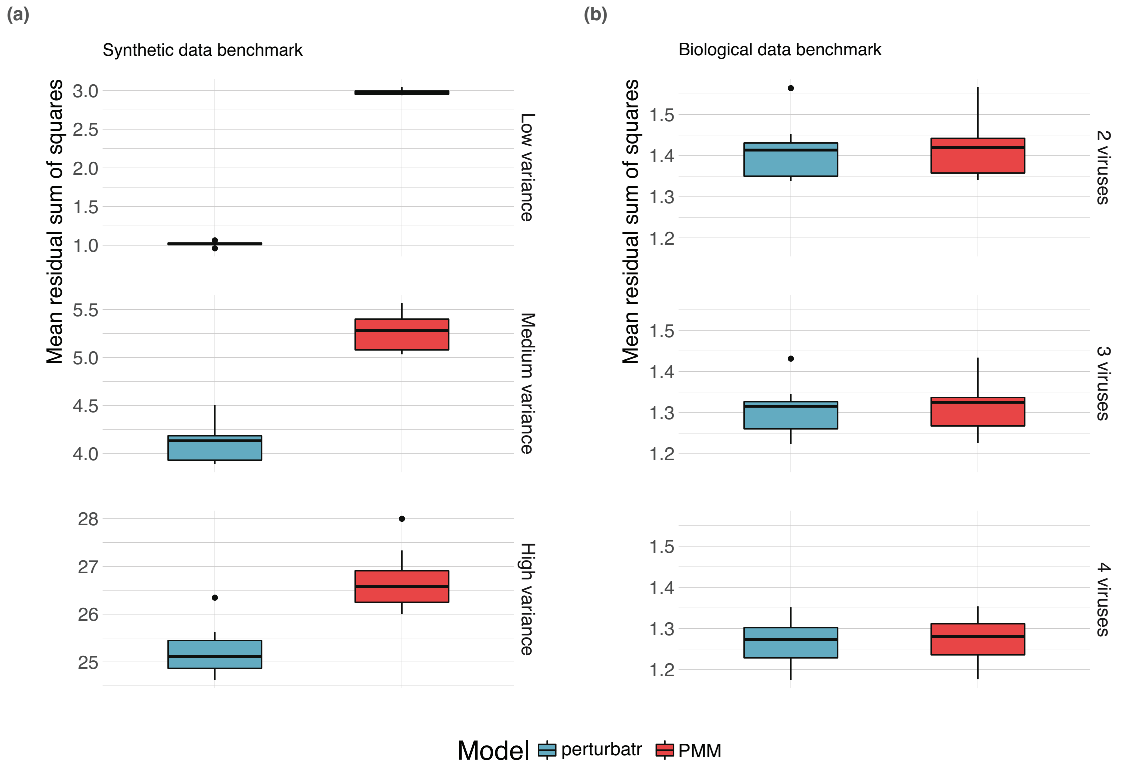

Supplement: S4 Fig — The plots show the performance of our hierarchical model against PMM. (PDF) [file pcbi.1007587.s012.pdf]
