# Supplementary material for: Host factor prioritization for pan-viral genetic perturbation screens using random intercept models and network propagation
Source: PLoS Comput Biol. 2020 Feb 10;16(2):e1007587. doi: 10.1371/journal.pcbi.1007587 (PMC7034926; doi:10.1371/journal.pcbi.1007587)

S5 Figure. Identification of host factors using a random effects model. The 25 first hits identified using the first step of our model are shown when sorting the estimates by absolute effect sizes. The size of the bar determines the ”pan-viral” effect a knockdown has on the entire group of pathogens. Knockdown of antiviral genes (in red) will generally increase pathogen viability, while pro-viral hits (in blue) will reduce it.

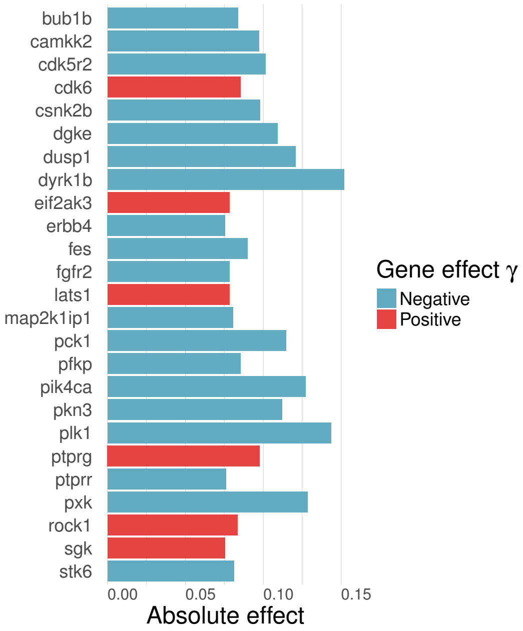

Supplement: S5 Fig — The 25 first hits identified using the first step of our model are shown when sorting the estimates by absolute effect sizes. (PDF) [file pcbi.1007587.s013.pdf]
