# Supplementary material for: Host factor prioritization for pan-viral genetic perturbation screens using random intercept models and network propagation
Source: PLoS Comput Biol. 2020 Feb 10;16(2):e1007587. doi: 10.1371/journal.pcbi.1007587 (PMC7034926; doi:10.1371/journal.pcbi.1007587)

S6 Figure. Validation of identified host factors using pharmacological inhibition. We screened the literature for compounds that have an inhibitory effect on the proteins of genes found in Table 2. Then, as described in Supplement S4 Text, we screened compounds available for purchase and measured cell viability (CHIKV, CVB, MERS) or virus replication (DENV, HCV), respectively. For CHIKV, CVB and MERS we tested if cell viability increases significantly after compound addition (one-sided two-sample Wilcoxon-test for each compound-concentration-virus combination with each 8 samples for CHIKV and MERS and 4 samples for CVB per test and side). For DENV and HCV we tested if addition of a compound reduced virus production (one-sided two-sample Wilcoxon-test for each compound-concentration-virus combination with 12, or 8 samples per test and side, respectively). Each significant test at a level of $\alpha = 5\%$ is highlighted with a blue dot indicating an anti-viral effect of the compound. Smallish, black dots indicate either non-significant p-values or discarding of the test due to compound cyto-toxicity (reduction to 75% cell viability) at the specified concentration. While no compound/gene has an overall inhibitory effect on virus replication, some compounds show effects for subsets of these viruses. All respective analyses can be found in Supplement S1 Code and the data in S1 Data.

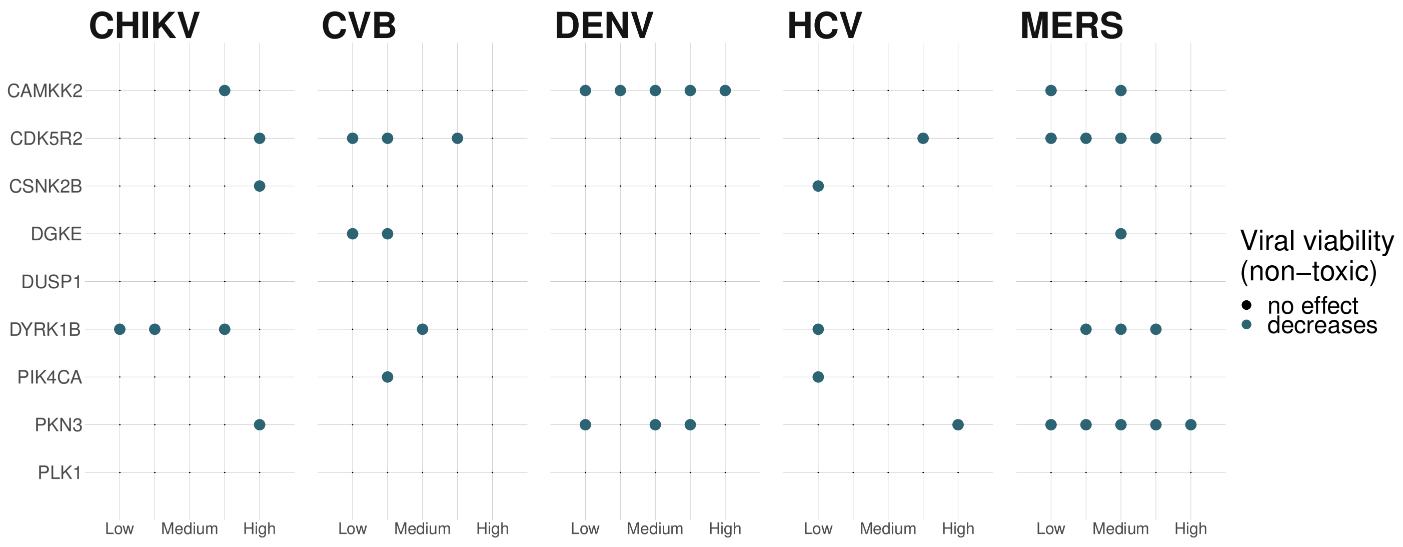

Supplement: S6 Fig — Description and visualization of the analysis of the validation screen using pharmacological inhibition. (PDF) [file pcbi.1007587.s014.pdf]
