# Supplementary material for: Host factor prioritization for pan-viral genetic perturbation screens using random intercept models and network propagation
Source: PLoS Comput Biol. 2020 Feb 10;16(2):e1007587. doi: 10.1371/journal.pcbi.1007587 (PMC7034926; doi:10.1371/journal.pcbi.1007587)
Supplement: Attachment — Submitted filename: responses.pdf [file pcbi.1007587.s015.pdf]

Comments to the Authors:
Please note here if the review is uploaded as an attachment.

Reviewer #1: In this work, the authors have proposed a two-stage method that combines a statistical model and network propagation to prioritize host factors from heterogeneous and noisy RNAi screens for four different viruses. First a statistical model based on random effects model is used to rank the genes by their absolute effect size. In the second stage, a network propagation approach based on protein-protein interactions has been employed to study the effect of these genes and further fine tuning of list of prioritized genes that have significant impact on pan-viral life-cycle. The manuscript has been written nicely and the different methods and discussions are well-understandable. Although, there is not much computational novelty, still the computational work is supported with biological validation which is the key contribution of it. I have following comments on the manuscript.

1. The authors have demanded that their work is on pan-pathogen level. However they have used data for four viruses in this work. Is there any particular reason of choosing the four viruses? Do the authors expect similar results for other virus or pathogen combinations?

We would like to thank the reviewer for this question. The reason for choosing the four viruses in the first place was the high similarity in their replication cycles. We hypothesized that the higher the biological similarity the more likely are the same host factors, or at least pathways, used for replication. Consequently, the success rate with other pathogens should depend on their similarity as well. For bacteria for instance, antibiotics also only work for a very similar group of bacteria (such as gram-positives). We added respective additions in the paper in the introduction as well as in the discussion.

2. While describing the random effects model, the authors have used mathematical notations. It is understood that they have used some R package for this work, however, very limited description is there on how this is actually implemented, how the data sets are organized, or how the data set is fit into the model. These descriptions will beneficial for the readers.

We thank the reviewer for this valuable comment. We added more information for clarification on the mathematics in the supplement. Since estimation of mixed modes is still an open question in research we illustrate the general approach for estimation of parameters and not the very complex approach from the R package lme4.

3. An important stage of this work is the use of functional protein-protein interaction network in the second stage. However, there is practically no description of the corresponding data. The authors have just referred to the publication (ref. [39]) from which they have collected the data. However a short description is required for the sake of complete understanding. Another concern is that the data set is pretty old (2010). They should have used more recent interaction data instead.\

The reviewer is right in referring to the choice of a fairly old network. We added some information in the method part why we think this network is still a good choice. The main reason is that the network consists of functional interactions, and not merely physical ones, and the fact that large parts of the network are expert-curated. Since the edges encode functional similarity, we are certain that connected genes are in the same protein complex or involved in the same pathway. Since viruses often do not use the exact same host factors but target the same pathways at different entry points, we hypothesized that a network like this is ideal for our setting. The fact that it was able to generate two novel host factor corroborates this hypothesis. To the best of our knowledge, similar, more modern networks of the same quality either do not exist, or if they exist the edges do not have a functional interpretation.

4. There are few typos such as follows:
-- Line 230: The models described by Equation (1) and Equation (3) estimates gene effects --> ... estimate ...
-- Use either 'knockdown' or 'knock-down' consistently.

Thank you! We corrected the typos.

Reviewer #2: The paper by Dirmeier et al. develops computational/statistical methods to identify host factors that are required by viruses for their replication, and that can be targets of therapeutic interference. While this approach has been done in select settings, such approaches to identify host factors on a viral group level are lacking. Identifying common host factors among a viral group would be desirable because this could allow the development of antivirals

with broad-spectrum activity. A maximum likelihood approach using a random effects model was employed, and this information was propagated over a biological graph using network diffusion with Markov random walks. This method was able to reproduce previous work that identified host factors for single viruses and was then used to predict host factors across pathogen groups. This was validated by inhibition experiments.

This is an important topic and analysis, and the methodology seems well-developed and sound. I have some relatively minor comments:

- Statistical approaches to identify important host factors have been used in other settings, as outlined in the introduction of the paper (e.g. for single viruses, two viruses of the same genus, etc). Have these approaches been successful in developing treatments? If so, this could be summarized in the introduction. If not, this could be reviewed, and relevance for developing treatments as a result of the novel methodology could be discussed. Such additions would be useful for a more general computational biology readership.

We thank the reviewer for these excellent comments that helped us improve the manuscript. To our knowledge pan-pathogen treatment has received only very little attention even though inhibitors for multiple viruses exist. We added an appropriate summary of existing approaches to the introduction.

- When identifying host factors that are relevant for a broad group of viruses, how do those factors that you identified compare with the factors that were identified in previous work in the context of more restricted settings? If a host factor is important for a broader group of viruses, is it likely that those host factors are more crucial for host cell function? If so, would that pose a problem to target them therapeutically? Perhaps a discussion of this could be added.

Generally, host factors are not necessarily important for the cells. However, for a broader group of viruses the inferred host factors tend to be central biological players such as UBC which are involved in multiple biological processes. However, treatments for these processes, for instance for cancer already exist and are used clinically. In such cases a fine balance between drug cytotoxicity and pan-viral efficacy has to be found. We added a respective paragraph to the discussion part of the manuscript.

- The paper reports that no host factor could be found that is significant for all viruses in the group under consideration (+ ssRNA viruses). It could be useful to discuss the implications of this. Is it likely a general result that holds if you look at a different group of viruses? What are the limitations in the breadth of the viruses that can be treated by targeting a given host factor?

For different groups of viruses, biological similarity of the replication cycle is the determining factor. The higher the similarity the more likely is it to find the same genes as host factors or at least the same pathways. Viruses usurp very defined and distinct host cellular pathways and proteins; even closely related viruses may use different routes due to differences in their molecular virology and structure of their proteins. The broader the group of viruses, the more central a target gene would have to be (e.g. UBC), but in fact, in that case, it might get more and more unlikely to find a inhibitor condition that only harms the virus and not the host cell. So the hope is that the closer a group of viruses is related, the higher the chance that they rely on the same specific pathway (e.g., a specific ubiquitin ligase system could be essential, so one would not have to inhibit at the very central level of ubiquitin itself). We added a paragraph with these considerations to the discussion.
